# Autofluorescence Imaging in the Long-Term Follow-Up of Scleral Buckling Surgery for Retinal Detachment

**DOI:** 10.1155/2022/2119439

**Published:** 2022-02-27

**Authors:** Panagiotis Salvanos, Helgi D. Björnsson, Valeria Vitelli, Ragnheiður Bragadόttir, Morten C. Moe, Tor P. Utheim

**Affiliations:** ^1^Department of Ophthalmology, Drammen Hospital, Vestre Viken Hospital Trust, Drammen, Norway; ^2^University of Oslo, Oslo, Norway; ^3^Department of Ophthalmology, Oslo University Hospital, Oslo, Norway; ^4^Oslo Centre for Biostatistics and Epidemiology, Department of Biostatistics, University of Oslo, Norway; ^5^Department of Medical Biochemistry, Oslo University Hospital, Oslo, Norway

## Abstract

**Purpose:**

To analyse fundus autofluorescence (AF) changes in retinal reattachment following primary scleral buckling (SB) surgery for rhegmatogenous retinal detachment (RRD).

**Methods:**

Prospective noninterventional chart review study. AF images were reviewed for peripheral and central changes and compared to clinical and OCT findings.

**Results:**

A total of 73 eyes from 69 patients were included, four presenting with bilateral RRD. Mean age was 55 ± 12 years, male/female ratio 40/29, fovea-on/-off RRD 43/30, and mean follow-up time 376 ± 270 days, with a mean of 5 ± 3 postoperative visits. Preoperatively, RRD was seen as a hypofluorescent area with a hyperfluorescent leading edge. Immediately postoperatively, three types of cryopexy could be differentiated, gradually transforming to scleral hyperfluorescence. Buckle tightening produced alternating hyper-/hypofluorescent streaks, and demarcation lines showed a persistent rugged hyperfluorescent signal. Choroidal detachment led to transient hypofluorescence, whereas vortex vein compression induced persistent hypofluorescence. Peripheral retinal folds were hyperfluorescent and the drainage site was hypofluorescent. AF was highly sensitive in detecting even small amounts of hyperfluorescent persistent subretinal fluid (SRF) that showed a slow resolution during follow-up. A granular “salt-and-pepper-” like pattern in the central macula was seen in 80% of eyes with fovea-off RRD and alternating streaks in 10%. Findings from OCT imaging correlated well with AF regarding SRF, macular oedema, retinal pigment epithelial detachment, and presence of a subretinal scar, but only moderately in epiretinal membrane formation and choroidal folds.

**Conclusions:**

AF is a useful, noninvasive, adjuvant tool in the long-term follow-up after SB surgery.

## 1. Introduction

In rhegmatogenous retinal detachment (RRD), the separation of the neural retina from the retinal pigment epithelium (RPE) leads to loss of the metabolic supply and cell death of the photoreceptors [1]. The preservation of vision requires surgical reapposition between these two layers, by means of either scleral buckle (SB), pars plana vitrectomy (PPV), or pneumatic retinopexy [2]. In recent years, the percentage of patients operated upon with SB has significantly decreased in favour of vitrectomy [3], with SB being today the method of choice in younger phakic patients with peripheral breaks and no major media opacities [4]. The induced refractive error and the compression of the arterial and venous supply causing metabolic alterations in the choroid and retina, together with the need for conjunctival peritomy, are the most discussed disadvantages of this technique [5, 6].

The metabolic stress induced on the retina by the detachment and the following surgical intervention can be observed by mapping fundus fluorophores [7]. These naturally autofluorescent substances can be imaged with fundus autofluorescence (AF), a noninvasive modality that provides an indirect marker of the metabolic activity of the outer retina and the balance between photoreceptor outer segment turnover and RPE phagocytotic ability [8]. Novel widefield technology has facilitated imaging up to 200° of the retinal periphery, where the success of a correctly placed SB lies. In addition, AF can be combined with other imaging modalities, such as angiography and optical coherence tomography (OCT), increasing our understanding of the pathophysiology of retinal detachment and reattachment [9]. Changes in retinal microvasculature, as seen in OCT angiography, can function as a predictor of surgical outcomes in macular surgery [10].

It has been previously published that AF can offer valuable insight in the immediate postoperative period after SB surgery [11]. Little is known, however, as to how these changes develop over time and what their correlation with clinical results is. In this study, we analysed the AF changes seen in the long-term postoperative follow-up of patients after SB and compared them to clinical findings and other imaging modalities, aiming to further improve our understanding of the pathophysiology of retinal reattachment after SB surgery for RRD.

## 2. Methods

The study was a noninterventional prospective chart review of patients undergoing primary scleral buckling surgery for RRD by one of the authors (P.S.) at the Department of Ophthalmology, Oslo University Hospital, Norway. The study was approved by the institutional data protection officer, and informed consent was obtained from all patients.

All patients were operated upon with a 2.5 mm encircling band, a 6 or 7 mm silicone segmental buckle, transscleral cryopexy, and the choice of diathermy drainage and air/SF6 gas tamponade. The exclusion criteria included previous vitreoretinal surgery, dense media opacities that obscured the view of the fundus, and inability to attend follow-up visits.

The follow-up period at the hospital was individually planned for each patient according to clinical indication. At all visits, every patient underwent a comprehensive ophthalmological examination, including best-corrected visual acuity (BCVA) by Snellen decimal units, applanation tonometry, slit-lamp biomicroscopy, and indirect ophthalmoscopy. The clinical examination was complemented with colour and AF fundus imaging and spectral-domain optical coherence tomography (Nidek Inc., Fremont, CA, USA). Upon indication, a subgroup of 13 patients additionally underwent ultra-widefield fundus fluorescein angiography (FFA) to evaluate the retinal circulation.

The ultra-widefield AF images were obtained using an Optomap P200Tx fundus camera (Optos, Dunfermline, UK) from both eyes during follow-up visits. A 532 nm (green) laser was used to capture ultra-widefield AF images, while composite colour images were obtained with the combined 532 nm and 633 nm laser during the same consultation. All AF images were subsequently transferred to ImageJ open-source software (version 1.52i, National Institutes of Health, USA) for image analysis.

The AF images were analysed for the presence of qualitative and quantitative changes. The qualitative analysis focused on a predefined set of descriptions for the effect of cryopexy, indentation of the SB and the encircling element, and macular changes. The cryopexy scar was classified as type 1 (a hyperfluorescent signal), type 2 (a central hypofluorescent area accompanied by a peripheral hyperfluorescent ring), type 3 (a completely hypofluorescent signal over the whole area), or scleral hyperfluorescence. The presence of subretinal fluid (SRF), alternating hyper-/hypofluorescent streaks on the segmental buckle and the encircling band, peripheral retinal folds, and choroidal detachment-induced hypofluorescence were documented as previously published [11]. In the macula, the presence of granular “salt-and-pepper” changes, alternating hyper-/hypofluorescent streaks, SRF, or scar was noted and controlled for possible correlation to clinical findings.

The subsequent quantitative image analysis was performed by transferring the uncompressed images to ImageJ. In the periphery, the cryopexy area was manually marked, and the pixel signal intensity analysed recording the area, signal intensity (recorded as average, median, minimum-maximum value, and standard deviation), and perimeter of the lesion. In the macula, we analysed the signal intensity in a circular area manually centred at the umbo with a 500-pixel diameter. In the vast majority of patients, this area diameter included the whole macula extending just within the temporal vascular arcades. A corresponding analysis was also performed for AF changes occurring only in the fovea by utilizing a smaller circle with a 150-pixel diameter centred manually at the umbo. The data recorded in both instances aimed at characterizing AF signal intensity (described as average, median, minimum-maximum value, and standard deviation of the signal).

All OCT images were qualitatively evaluated for the presence of persistent SRF postoperatively, cystoid macular oedema (CMO), epiretinal membrane formation (ERM), choroidal folds, focal irregularities of the ellipsoid zone (EZ), and the presence of a subretinal scar. These results were then compared to the clinical and AF findings after surgery.

The 488 nm blue laser of the Optomap P200Tx was used for image acquisition and ImageJ software for image analysis. The qualitative analysis focused on the presence of CMO, peripapillary dye leakage, peripheral demarcation line(s), alternating hyper-/hypofluorescent streaks on the buckle, visible sclerochoroidal drainage site, and peripheral vascular leakage (PVL), qualitatively classified on a 0–4 scale. In addition, qualitative analysis of the cryopexy lesions was carried out following manual marking, recording the area (in pixels), perimeter, and signal intensity.

The statistical analysis used to check for possible correlation between the AF, clinical, OCT, and FFA imaging findings was performed using R statistical software (R Core Team (2019), Vienna, Austria). A mixed-model analysis using AF imaging variables in the macula (defined as the whole macular area or only the foveal area) or the periphery as outcome, clinical data (fovea-on/off RRD, detachment size, BCVA, number of operations, and time), OCT, and FFA findings as predictors was performed.

## 3. Results

A total of 73 eyes from 69 patients were included in the present study. The preoperative and the demographic features are listed in Table S1. Four patients (5.8%) presented with bilateral RRD and were operated upon with SB in both eyes in sequential surgeries. Mean follow-up time was 376 days (standard deviation, 270 days; range, 30–2711 days; median, 282 days), with a mean of 5 ± 3 postoperative visits (range, 2–19 visits). The postoperative course of the patients is listed in Table S2 (see Table S2a for changes in refraction and Table S2b for the clinical course following surgery).

### 3.1. Retinal Imaging Findings

#### 3.1.1. Autofluorescence Findings

*(1) Preoperative*. The RRD was apparent preoperatively in AF images as a hypofluorescent area with a hyperfluorescent posterior border at the leading edge of the detachment. Large retinal breaks were easily identified as hyperfluorescent areas due to window defect. Small breaks were difficult to identify in AF imaging.

*(2) Postoperative*.

**Figure 1:**
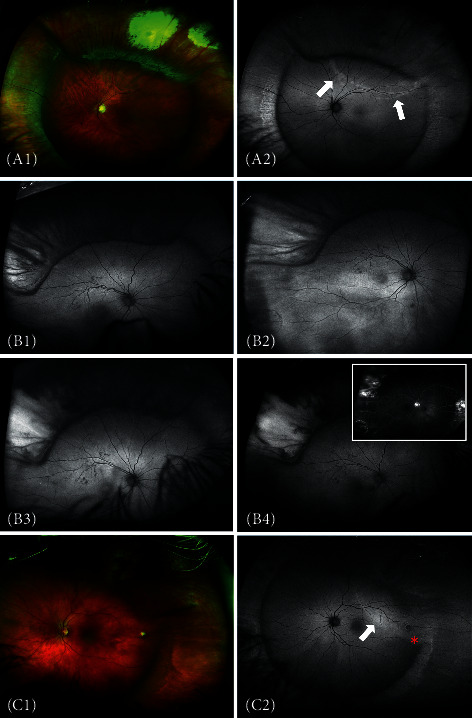
Peripheral autofluorescent (AF) retinal changes after scleral buckling surgery. (A1-2) Colour and autofluorescent imaging showing peripheral demarcation line (white arrows). No change during follow-up was seen. Note more pronounced changes in AF. (B1–4) Evolution of the cryopexy scar 1, 2, 8, months and 2, 5 years postoperatively seen with autofluorescence. The scleral hyperfluorescence remained unchanged after 8 months. Corresponding fluorescein angiography (B4 insert). (C1-2) Low subretinal fluid behind the buckle, difficult to detect ophthalmoscopically (colour image-C1), but visible hyperfluorescence in AF (C2). Note a hypofluorescent spot corresponding to the scleral drainage site (red asterisk-C2).

**Figure 2:**
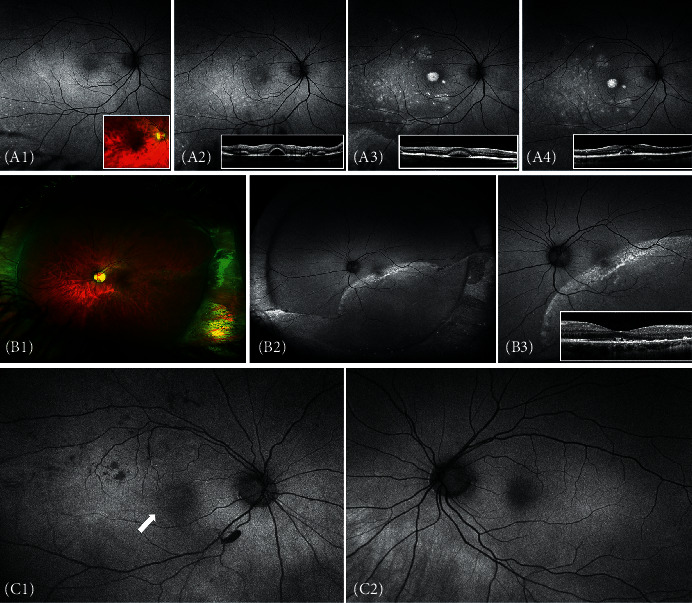
Macular autofluorescent changes after scleral buckling surgery. (A1–4) Evolution of persistent subretinal fluid after 1 day (A1, colour image insert), 45 days (A2), 6 months (A3), and 1 year (A4) after surgery. Corresponding OCT images (inserts). (B1–3) Alternating streaks after reattachment in colour (B1) and autofluorescent imaging (B2 and high-resolution image B3). Corresponding OCT (insert); note the outer retina microfolds. (C1-2) Granular “salt-and-pepper-” like changes in AF imaging in the right eye (white arrow in C1) compared to the collateral eye of the same patient (C2).

  Periphery: postoperatively, we observed peripheral alternating hyper-hypofluorescent radial streaks on the tightened encircling band in 44 eyes (60%) and corresponding changes on the segmental buckle in 27 eyes (37%). No significant change in the number or signal intensity of these streaks was seen during follow-up.
  There was obvious peripheral hyperfluorescence from persistent SRF in a total of 19 eyes (26%) after surgery (Figure 1). Of these, the peripheral hyperfluorescence disappeared between the first and second postoperative appointment (mean, 49 days; range, 5–111 days) in 11 eyes (58%), whereas in the remaining eight eyes (42%), the hyperfluorescent signal remained visible, although of gradually decreasing intensity, throughout the follow-up (mean, 239 days, range, 31–519 days).
  A peripheral hyperfluorescent retinal fold was evident in four eyes (5.5%) postoperatively. In two eyes, the retinal fold appeared straight after successful reattachment, and the hyperfluorescent signal in AF imaging remained throughout the follow-up period. One eye had a retinal fold that disappeared 3 months postoperatively, and another eye developed several folds after PPV due to redetachment. The AF findings correlated very well with ophthalmoscopy.
  Eleven cases (15%) had one or several peripheral demarcation lines that showed a rugged hyperfluorescence and were readily visible in AF imaging throughout the follow-up with no observable change. The sclerochoroidal drainage site was ophthalmoscopically evident in 45 eyes (62%) as a hypofluorescent spot, sometimes accompanied by a thin hyperfluorescent halo (Figure 1).
  Eight eyes (11%) developed choroidal detachment that produced a hypofluorescent signal during the immediate postoperative period. There was significant discrepancy in the duration of the hypofluorescent signal, with five cases disappearing within the first postoperative month, two cases disappearing within three months, and one case lasting for almost 9 months with complete normalization thereafter. Four eyes (5.5%) showed postoperative hypofluorescence from a swollen vortex vein. This finding was consistent during the whole follow-up period showing no change in size or signal intensity.
  When analysing the cryopexy effect in the immediate postoperative period, seven eyes (9.5%) were found to have type 1, 20 eyes (27.5%) type 2, and the remaining 46 eyes (63%) type 3 cryopexy effect. All eyes with type 1 and 2 cryopexy effect changed over time to type 3, which in turn gradually changed to scleral hyperfluorescence following the development of chorioretinal atrophy in the treated area, corresponding well with ophthalmoscopic findings and colour fundus images (Figure 1).
  Macula. The qualitative analysis of the postoperative AF images (Table 1) revealed a granular “salt-and-pepper-” like pattern in 24 eyes with fovea-off RRD (80%), as well as in nine eyes with fovea-on RRD (21%). Alternating hyper-/hypofluorescent streaks were found in only three eyes (10%) with fovea-off RRD. Another eye with fovea-on RRD with no visible macular changes in AF after SB developed a secondary ERM and after PPV and peeling presented visible alternating streaks on AF. Hyperfluorescence from persistent SRF in the macular area was readily visible postoperatively in one eye (2%) with fovea-on RRD and in 16 eyes (53%) with fovea-off RRD. The resorption of the SRF over time was illustrated clearly in the AF images, with a transformation of the diffuse hyperfluorescence into a number of hyperfluorescent lobules, with a subsequent decrease in the SRF pocket size and number (Figure 2). From these eyes, seven (23%) with fovea-off RRD showed a combination of hyperfluorescent SRF and granular “salt-and-pepper-” like changes in AF imaging. One eye with fovea-off RRD showed an alternating hyper-/hypofluorescent macular scar that persisted throughout the follow-up.
  The mixed-model analysis (Table 2) revealed a statistically significant negative correlation (*R*^2^ = 35.9%; Fisher's test for regression *P* value = 0.00024) between the mean AF pixel value in the macular area and the size of the retinal detachment (*P*=1.2%, 95% CI = [−0.49, −0.07]) and the number of operations (*P*=4.1%, 95% CI = [−0.45, −0.01]), respectively. The significant predictor was time (*t*-test for the single predictor *P* value = 0.00038). The residuals were Gaussian (Shapiro test for normality *P* value = 0.08). No statistically significant correlation was found between the AF signal in the fovea or the periphery and other clinical variables.

#### 3.1.2. OCT Findings and Comparison to AF Imaging

The OCT examination revealed EZ irregularities in five eyes (6.8%), showing good correspondence with AF findings in four of five eyes (80%) (Table S3). An ERM was seen postoperatively in 12 eyes (16%), with only half of them demonstrating corresponding changes in AF. Choroidal folds were observed in one eye (1.3%) but were not visible in AF. OCT imaging revealed SRF in 13 eyes (18%), with 12 of them exhibiting a hyperautofluorescent signal. One eye (1.3%) had RPE detachment (PED) in OCT and granular changes in AF. Five eyes (6.8%) had CMO in OCT imaging while showing various AF changes. Finally, one eye (1.3%) presented postoperatively with a subretinal scar and corresponding AF granular changes. Altogether, although good correspondence in detecting pathology was found with both OCT and AF, we were not able to identify a correlation between specific hypo- or hyperfluorescent AF patterns and OCT changes.

#### 3.1.3. FFA Findings

A total of 13 patients included in this study underwent FFA imaging upon indication. Ten patients were subjected to one angiogram, one underwent two examinations, and another patient underwent four angiograms during follow-up. The patient receiving two FFA had preoperatively a peripheral retinal haemangioma in the detached retina that was attributed to chronic retinal detachment. The haemangioma disappeared after focal transscleral cryotherapy during the SB procedure. The patient that was subjected to four angiograms due to the development of progressive retinal ischemia with neovascularization in both eyes during follow-up after SB for RRD, combined with neurosensory hearing loss and neurological symptoms, was diagnosed with possible Susac's syndrome and was treated accordingly with panretinal photocoagulation, systemic steroids, and immunosuppressant therapy, with a good outcome as previously reported [12].

**Figure 3:**
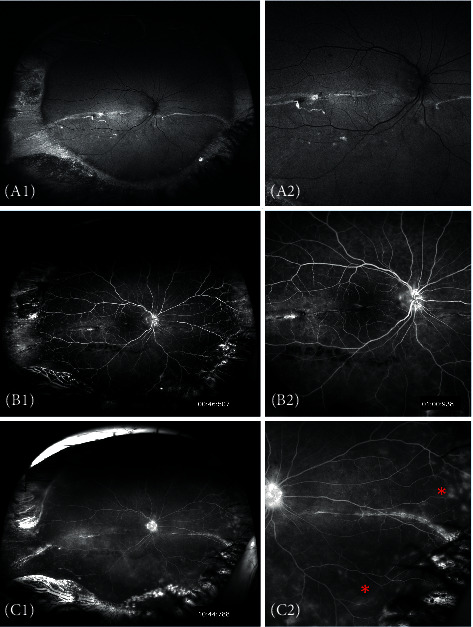
Autofluorescent (AF) and fluorescein angiography (FFA) findings in a patient with macular scar after reattachment. (A1-2) Hyper-/hypofluorescence changes in AF imaging. (B1-2) Less pronounced changes centrally in FFA imaging. (C1-2) Extensive peripheral vascular leakage in the late angiographic phase of the same patient with multiple leaking points in the periphery (C2 red arrows). Note that these changes were not visible in AF imaging.

Overview of the imaging findings in FFA and corresponding OCT and AF findings are presented in Table 3. The overall correspondence between the FFA images and AF images in the periphery was good in all 13 patients tested. All cases had obvious alternating hyper-/hypofluorescent streaks on the encircling band and the segmental buckle, matching the alternating streaks seen in the AF images. In FFA, the drainage site was evident in eight eyes (61.5%) as a hyperfluorescent spot, whereas in AF the same lesion was hypofluorescent in all 13 cases (100%). A demarcation line was easily identified in three eyes (23%) and correlated well with AF, although it was only obvious in AF imaging and not FFA in another patient. A statistically significant correlation (*P*=0.03) was found between the size of the peripheral cryopexy lesion in FFA and AF imaging. Five eyes (38.5%) showed no or minimal PVL, and four eyes significant widespread PVL. Interestingly, AF was not sensitive in detecting PVL (Figure 3).

In the macula, there was discrepancy between the different imaging modalities. Angiographic CMO was observed in four eyes. In each of these eyes, OCT showed different pathology; intraretinal oedema, subretinal SRF, ERM development, and normal tomographic anatomy. On AF imaging, we could identify granular changes in only two of these eyes. Two eyes had angiographic late leakage from the optic nerve without any clinical signs of optic disc oedema, combined with significant PVL. AF imaging in these eyes showed no corresponding changes on or around the optic nerve. Interestingly, from the nine cases that did not reveal any pathology in the posterior pole on FFA imaging, AF showed macular changes in four cases, and OCT showed macular changes in two additional cases (one case had missing examination data) (Table 3).

## 4. Discussion

To our knowledge, this is the first study to use ultra-widefield fundus AF to analyse metabolic stress patterns and functional recovery of the retina and the RPE in long-term follow-up after scleral buckling surgery.

The anatomical success of the surgery relies on a correctly placed buckle with the proper indentation and retinopexy. The ensuing metabolic alterations induced by the buckle have been so far studied by demanding methods, not integrated in everyday clinical practice (such as angiography, laser speckle, and laser Doppler technology) [5, 13]. Postoperative visual acuity depends on photoreceptor and RPE regeneration after successful reattachment. Multifocal electroretinography has been previously employed to study these changes but is not widely used due to the fact that it is time-consuming. On the other hand, modern OCT can depict the microanatomy in great detail but without revealing the metabolic stress exerted on the photoreceptor-RPE complex [14–16]. Ultra-widefield fundus AF opens new possibilities in the study of both peripheral and central changes [17]. The use of green excitation wavelength (532 nm) that is not absorbed by the macular pigment as blue light makes the analysis of macular changes easier.

The improvement in BCVA and the refractive changes observed after surgery are in accordance with previously published results [18]. The positive correlation that we observed between the change in spherical value and RRD size can be explained by a tendency for a tighter buckle resulting in higher myopic shift in more longstanding retinal detachments. The redetachment rate (4.1%) is in agreement with previous reports, with successful reattachment after a supplementary retinal procedure in all patients [4].

Preoperative AF findings confirmed previous published findings [11]. The postoperative alternating hyper-/hypofluorescent streaks over the explants due to scleral indentation and RPE/retinal microfolds proved to be a lasting finding, indicating no measurable change in the microanatomy of the area compressed by the SB. Corresponding alternating hyper-/hypofluorescent streaks were also identified in the subgroup of patients imaged with FFA.

Even small quantities of SRF postoperatively were easily identified in AF imaging as a strongly hyperfluorescent area, a highly specific finding, with a distinct pattern of reduction over time in both the periphery and the macula. This can be helpful in differentiating persistent SRF from other entities that may indicate the need for a new intervention, such as shallow redetachment in the periphery that has a characteristic pattern in the leading edge of the detachment [19] or CMO in the macula. Over time, the initial hyperfluorescent area reduced in size, changing subsequently into a number of distinct hyperfluorescent lobules that gradually decreased in size and number before disappearing. The timespan needed for complete SRF absorption varied significantly between patients, a finding attributed to varying degrees of RPE pump function and significantly different SRF viscosity due to the duration of the detachment.

Autofluorescence imaging with AF was also very sensitive in revealing demarcation lines, a finding that persisted during the follow-up. Intriguingly, in the small subgroup of patients who underwent FFA, AF was more sensitive in depicting demarcation lines compared to the angiogram. Hyperfluorescent peripheral retinal folds in AF also corresponded well with clinical findings and remained unchanged during follow-up as long as the folds were clinically visible. Further, we found that postoperative hypofluorescence can be differentiated due to choroidal detachment or vortex vein stasis. Choroidal detachment was found to lead to a marked hypofluorescent signal that normalized over time (though with a significant timespan between patients) correlating well with ophthalmoscopic findings, whereas the hypofluorescent signal over congested vortex vein(s) remained unchanged during the follow-up.

While scleral buckling surgery has proven to be an effective method for treating RRD, anatomical failure due to PVR remains a concern, with the amount of cryopexy used strongly implicated in PVR development [20, 21]. Here, we used AF to follow up the cryopexy lesions over time in an attempt to objectively quantify the pathophysiological processes and improve vitreoretinal surgical feedback. By utilizing a previously published classification system for the cryopexy effect as seen in AF imaging [11], we observed a gradual change of the AF signal in all cases to scleral hyperfluorescence, corresponding to the ensuing chorioretinal atrophy seen clinically and in accordance with histological and clinical studies showing gradual atrophy of the retina and RPE with increasingly visible sclera [21]. The timespan for these AF changes varied substantially between the patients, being shorter when hypofluorescence was seen in the immediate postoperative period, due to application of more intense cryopexy around the break. Our long-term follow-up data indicated that no change was seen after the development of scleral hyperfluorescence. Statistical analysis of the AF images revealed a tendency to use more intense cryopexy in eyes presenting with worse BCVA. We also found strong statistical correlation between cryopexy size in AF imaging, ophthalmoscopy (as documented and quantified in colour fundus images), and FFA (in the subgroup tested), suggesting that AF can be utilized as an objective method for documenting, classifying, and following up the extent of RPE disruption after retinopexy. Further research with a larger sample size is needed to confirm whether this finding can serve as a future early biomarker of PVR formation.

Peripheral vascular leakage (PVL) is an important angiographic finding in cases of uveitis, indicating peripheral vascular inflammation and leakage [22]. To date, it is not known if this angiographic finding correlates with the clinical course after retinal detachment surgery. Interestingly, PVL could not be detected in AF, showing that these two imaging techniques are not interchangeable but complementary in peripheral retinal evaluation. Corresponding findings were also seen in cases of dye leakage in the macula and around the optic nerve. We speculate that this is due to the different source of signal between these two techniques, with FFA focusing on the circulation and leakage of the dye from the vasculature, whereas the main source of AF is LF at the RPE-photoreceptor level.

The known anatomical success rate of conventional buckling surgery for RRD is good [3, 4], but visual recovery after successful reattachment can be limited by macular changes, such as CMO, microstructural changes in the EZ due to patchy recovery of the outer retinal layers, macular fold, or ERM. In fovea-off RRD, complete visual recovery is seldom achieved, even with an almost normal-appearing macula on clinical examination during follow-up [23]. OCT can identify many ultrastructural macular changes, and while they do correlate with decreased postoperative visual acuity and focal defects in retinal sensitivity [24], there is limited agreement between OCT and AF [25, 26]. Study of postoperative changes in the retinal microvasculature with widefield OCT angiography indicates increased metabolism following vitreoretinal surgery [10]. Both techniques have been proven sensitive for detecting partial-thickness retinal folds that occur after PPV for retinal detachment and can limit visual recovery [27]. In contrast to previous findings, we discovered outer retinal microfolds in only a small subgroup of eyes (10%), with a significantly higher prevalence of granular changes (fovea-off group, 80%; fovea-on group, 20%). We speculate that this may be due to the different SRF drainage mechanisms used during SB surgery; a slower and more passive removal of the SRF in SB surgery can lead to smoothening of preexisting outer retinal corrugations before complete reattachment compared to the active drainage mechanism of vitrectomy or a higher incidence of outer retinal macular folds in vitrectomy due to unintentional retinal displacement [28]. Moreover, the statistical analysis revealed a tendency for eyes presenting with larger retinal detachments involving the macula and eyes needing reoperations to exhibit a more hypofluorescent signal in the central macula during follow-up, indicative of potential lower metabolic activity of the RPE. Further research is required to confirm the importance of this finding.

Finally, only half of the eyes with ERM on OCT showed corresponding findings in AF, whereas in cases of CMO, persistent SRF, and subretinal scar the correspondence was good between these two imaging techniques. We hypothesize that these differences are due to the fact that AF photographs a dynamic process, depicting the metabolic stress at RPE-photoreceptor complex, whereas OCT displays the microstructure of the whole retina and choroid. We lack at present a prediction biomarker of the final visual acuity after retinal reattachment.

Limitations of the present study include the fact that fundus AF is a dynamic process and image quantification was performed manually due to lack of an automated system. The sample size was relatively small and with a variable follow-up period. Studies with a larger population size and combination with microperimetry testing to test point-to-point correspondence of the AF changes in the macula to light sensitivity can be helpful in delineating how AF changes correlate to functional outcomes after reattachment.

In conclusion, ultra-widefield fundus AF proves to be a useful noninvasive adjuvant tool in the examination and long-term follow-up of scleral buckling surgery. It offers valuable insight into the pathophysiological mechanisms of retinal repair and can serve as a potential biomarker after surgical treatment for RRD.

## Figures and Tables

**Table 1:** Overview of the qualitative autofluorescent changes seen in fovea-on and fovea-off rhegmatogenous retinal detachment after scleral buckling surgery.

	Autofluorescent changes:					
	No obvious changes	Hyperfluorescent SRF within the vascular arcades	Granular changes	Hyperfluorescent SRF and granular changes	Alternating hyper-/hypofluorescent streaks	Hyper-/hypofluorescent scar
Fovea-on detachment (total, 43)	33	1	9	0	0^*∗*^	0
Fovea-off detachment (total, 30)	3	9	17	7	3	1

SRF = subretinal fluid. ^*∗*^One eye was subjected to pars plana vitrectomy and peeling for a secondary epiretinal membrane and developed alternating hyper-/hypofluorescent streaks after the later surgery (not included in the table).

**Table 2:** Autofluorescent changes in the macula: analysis of variance for the fixed effects associated with the mixed model.

	Fovea-on/off	Detachment size	BCVA	OCT	Number of operations	Time
Macula mean (median, mode)	0.17 (0.20, 0.34)	0.39 (0.33, 0.15)	**0.0091 (0.015, 0.037)**	0.20 (0.19, 0.11)	0.094 (0.10, 0.089)	0.25 (0.21, 0.29)
Fovea mean (median, mode)	**0.031 (0.042**, **0.078)**	0.60 (0.64, 0.59)	0.23 (0.24, 0.075)	**0.036 (0.034**, **0.015)**	0.091 (0.098, 0.059)	0.17 (0.11, **0.014**)
Periphery mean (median, mode)	0.29 (0.39, 0.4)	0.89 (0.86, 0.91)	**0.002 (0.0003**, **0.00003)**	0.19 **(0.036**, **<10**^**−5**^**)**	0.061 (0.072, 0.41)	0.5 (0.31, 0.38)
Periphery area	0.95	0.86	**0.0022**	0.31	0.36	0.61
Periphery perimeter	0.86	0.57	0.06	0.98	0.53	0.91

Each line in the table corresponds to a different outcome, as indicated in the first column. Only *P* values are reported. Significant model/effects combinations are in bold. BCVA = best-corrected visual acuity, OCT = optical coherence tomography.

**Table 3:** Qualitative same-day fluorescein angiography, optical coherence tomography, and autofluorescence imaging findings in a subgroup of 13 eyes.

Nr	Foveal status	FFA in the macula		FFA in the periphery		OCT	AF in the macula			AF in the periphery		
		“Flower petal” leakage	Hot disc	Peripheral demarcation line	Degree of PVL		Granular changes	SRF	Alternating hyper–hypo-AF streaks	Scar	Peripheral demarcation line	Peripheral SRF
1	On	1	1	1	3	0	0	0	0	0	1	0
2	Off	0	0	0	0	0	0	0	0	0	1	0
3	Off	0	0	0	1	SRF	1	0	0	0	0	0
4	Off	1	1	1	4	SRF	1	0	0	1	1	0
5	On	0	0	1	0	0	0	0	0	0	1	0
6	Off	0	0	0	4	ERM	1	0	0	0	0	0
7	On	0	0	0	3	ERM	0	0	0	0	0	0
8	On	0	0	0	0	EZ changes	0	1	0	0	0	1
9	On	0	0	0	2	ERM	0	0	0	0	0	0
10	Off	0	0	0	4	SRF	1	0	0	0	0	0
11	On	0	0	0	2	(No data)	0	0	0	0	0	1
12	On	1	0	0	0	ERM	0	0	0	0	0	0
13	On	1	0	0	2	CMO	1	0	0	0	0	0

FFA = fundus fluorescein angiography, OCT = optical coherence tomography, AF = autofluorescence, EZ = ellipsoid zone, SRF = subretinal fluid, PVL = peripheral vascular leakage, ERM = epiretinal membrane, and CMO = cystoid macular edema.

## Data Availability

The raw patient data used to support the findings of this study have not been made available because of patient privacy. Unidentified imaging data used to support the findings of this study are included within the supplementary information files.

## Supplementary Materials

Supplementary Materials Table S1: preoperative and demographic characteristics of patients with rhegmatogenous retinal detachment. Table S2: postoperative changes after scleral buckle for rhegmatogenous retinal detachment: changes in refraction (Table S2a) and clinical course following surgery (Table S2b). Table S3: overview of the optical coherence tomography and autofluorescence findings.
