# Supplementary material for: Autofluorescence Imaging in the Long-Term Follow-Up of Scleral Buckling Surgery for Retinal Detachment
Source: J Ophthalmol. 2022 Feb 27;2022:2119439. doi: 10.1155/2022/2119439 (PMC8898876; doi:10.1155/2022/2119439)
Supplement: Supplementary Materials — Table S1: preoperative and demographic characteristics of patients with rhegmatogenous retinal detachment. Table S2: postoperative changes after scleral buckle for rhegmatogenous retinal detachment: changes in refraction (Table S2a) and clinical course following surgery (Table S2b). Table S3: overview of the optical coherence tomography and autofluorescence findings. [file 2119439.f1.zip › 2119439.f1/Table S1.docx]

***Table S1***

|  | **Preoperative clinical findings** |
| --- | --- |
| Total number of patients | 69 |
| Total number of eyes | 73 |
| Male/female ratio | 40/29 |
| Mean age (years) | 55 ± 12 (mean age male/female = 41/50 years) |
| Mean follow-up (days) | 376 ± 270 (range, 30-2711 days; median, 282 days) |
| Detailed follow-up duration | 2 eyes for 1 month, 9 eyes for 2 months, 8 eyes for 3 months, 7 eyes for 6 months, 18 eyes for 12 months, 12 eyes for 18 months, 11 eyes for 24 months, 4 eyes for 2 years, 2 eyes for 2+ years. |
| Fovea-on/-off RRD | 43/30 |
| RRD size (hours) | Mean 5 ± 2 (range, 1-12) |
| Right/Left eye | 38/35 |
| Lens status | 72 phakic eyes, 1 aphakic, 1 pseudophakic.  11 eyes (16%) with visually insignificant peripheral lens opacities |
| BCVA | 0.5 ± 0.4 (median, 0.6; range, 0.001-1.25) |
| Spherical value | Mean, -2.66 D ± 3.81 D (median, -2.00 D; range, -11.25 D to +2.50 D [phakic patients] and +11.00 D [the one aphakic patient]) |
| Astigmatism | Mean, -0.76 D ± 0.85 D (median, -0.50 D; range, plano to -4.25 D) |
| Fundus pathology in the contralateral eye | Peripheral retinal breaks in 9 eyes (13%).  Lattice degeneration with holes in 13 eyes (19%). |

Preoperative and demographic characteristics of patients with rhegmatogenous retinal detachment (RRD).

BCVA = best-corrected visual acuity.
