# Supplementary material for: Autofluorescence Imaging in the Long-Term Follow-Up of Scleral Buckling Surgery for Retinal Detachment
Source: J Ophthalmol. 2022 Feb 27;2022:2119439. doi: 10.1155/2022/2119439 (PMC8898876; doi:10.1155/2022/2119439)
Supplement: Supplementary Materials — Table S1: preoperative and demographic characteristics of patients with rhegmatogenous retinal detachment. Table S2: postoperative changes after scleral buckle for rhegmatogenous retinal detachment: changes in refraction (Table S2a) and clinical course following surgery (Table S2b). Table S3: overview of the optical coherence tomography and autofluorescence findings. [file 2119439.f1.zip › 2119439.f1/Table S2.docx]

***Table S2a***

|  | ***Postoperative clinical findings*** | ***P*-value** |
| --- | --- | --- |
| BCVA | 0.75 ± 0.24 (median 0.8, range 0.1-1.6) |  |
| BCVA gain | 0.26 ± 0.39 (median 0.25, range -0.9 to 1.25) | <0.001 |
| Sph | -4.31D ± 4.00D (median -3.36D, range -13.25D to +1.00D in phakic, +11.75D in the aphakic patient) |  |
| Sph change | -1.63D ± 1.22D (median -1.50D, range -4.50D to +1.50D) | <0.001 |
| Cyl | -1.40D ± 1.06D (median -1.25D, range 0 to -5.00D) |  |
| Cyl change | -0.60D ± 0.88D (median -0.75D, range -2.75D to +3.00D) | <0.001 |

Table S2a: Postoperative changes in the refraction of patients operated with scleral buckle for rhegmatogenous retinal detachment (RRD)

BCVA = best-corrected visual acuity, Sph = spherical value, Cyl = cylindrical value.

***Table S2b***

| ***Postoperative clinical course & complications:*** | ***Nr of eyes:*** | ***Type of intervention:*** |
| --- | --- | --- |
| Residual detachment in the immediate postoperative period | 2 (2.7%) | SF6 injection and laser retinopexy |
| Retinal re-detachment | 3 (4.1%)* | PPV with laser and gas tamponade |
| SB exposure | 3 | SB removal* |
| ERM development | 3 | PPV and peeling |
| Nuclear sclerosis with myopic shift | 3 | Phacoemulsification |
| Retinal ischemia and NV (Susac’s syndrome) | 1 | PRP and systemic immunosuppressants |

Table S2b: Postoperative clinical course after of patients operated with scleral buckle for rhegmatogenous retinal detachment (RRD)

SB = scleral buckle, SF6 = sulfur hexafluoride, SB = scleral buckling surgery, PPV = pars plana vitrectomy, ERM = epiretinal membrane, NV = neovascularization, PRP = panretinal photocoagulation.

* One eye developed re-detachment after SB removal.
