# Supplementary material for: Autofluorescence Imaging in the Long-Term Follow-Up of Scleral Buckling Surgery for Retinal Detachment
Source: J Ophthalmol. 2022 Feb 27;2022:2119439. doi: 10.1155/2022/2119439 (PMC8898876; doi:10.1155/2022/2119439)
Supplement: Supplementary Materials — Table S1: preoperative and demographic characteristics of patients with rhegmatogenous retinal detachment. Table S2: postoperative changes after scleral buckle for rhegmatogenous retinal detachment: changes in refraction (Table S2a) and clinical course following surgery (Table S2b). Table S3: overview of the optical coherence tomography and autofluorescence findings. [file 2119439.f1.zip › 2119439.f1/Table S3.docx]

***Table S3***

| **Foveal status preoperatively** | **OCT findings** | **Autofluorescence findings** | | | |
| --- | --- | --- | --- | --- | --- |
| On versus off | Type of postoperative pathology | Granular “salt-and-pepper” changes | Hyperfluorescent SRF | Alternating hyper-/hypofluorescent streaks | Scar |
| Off | Ellipsoid zone | + | - | - | - |
| Off | Ellipsoid zone | + | + | - | - |
| Off | Ellipsoid zone | - | - | - | - |
| On | Ellipsoid zone | - | + | - | - |
| Off | Ellipsoid zone | - | + | + | - |
| On | ERM | + | - | - | - |
| Off | ERM | + | - | - | - |
| Off | ERM | + | + | - | - |
| On | ERM | - | - | - | - |
| Off | ERM | - | - | - | - |
| Off | ERM | + | - | - | - |
| Off | ERM | + | - | - | - |
| On | ERM | - | - | - | - |
| Off | ERM | - | - | - | - |
| On | ERM | - | - | - | - |
| On | ERM | - | - | - | - |
| On | ERM | + | - | - | - |
| On | Choroidal folds | - | - | - | - |
| On | PED | + | - | - | - |
| On | SRF | + | - | - | - |
| Off | SRF | + | + | - | - |
| Off | SRF | + | + | - | - |
| Off | SRF | + | - | - | - |
| Off | SRF | + | + | + | - |
| Off | SRF | + | - | - | + |
| Off | SRF | + | - | - | - |
| Off | SRF | + | - | - | - |
| On | SRF | - | + | - | - |
| Off | SRF | - | + | - | - |
| Off | SRF | - | + | + | - |
| Off | SRF | + | - | - | - |
| On | SRF | - | - | - | - |
| On | PED | + | - | - | - |
| On | CMO | - | - | - * | - |
| Off | CMO | + | - | - | - |
| Off | CMO | - | + | - | - |
| Off | CMO | + | - | - | - |
| On | CMO | + | - | - | - |
| On | Subretinal scar | + | - | - | - |

Table S3: Overview of the optical coherence tomography (OCT) and autofluorescence findings. Normal OCT findings are not included in this table.

SRF = subretinal fluid, ERM = epiretinal membrane formation, PED = retinal pigment epithelial detachment, CMO = cystoid macular edema, PPV = pars plana vitrectomy.

* Not initially, but developed after PPV.
